# Obesity Hinders the Protective Effect of Selenite Supplementation on Insulin Signaling

**DOI:** 10.3390/antiox11050862

**Published:** 2022-04-28

**Authors:** Robert Hauffe, Michaela Rath, Wilson Agyapong, Wenke Jonas, Heike Vogel, Tim J. Schulz, Maria Schwarz, Anna P. Kipp, Matthias Blüher, André Kleinridders

**Affiliations:** 1 Department of Molecular and Experimental Nutritional Medicine, Institute of Nutritional Science, University of Potsdam, D-14558 Nuthetal, Germany; rhauffe@uni-potsdam.de (R.H.); rath@uni-potsdam.de (M.R.); wilson.agyapong@uni-potsdam.de (W.A.); 2 German Institute of Human Nutrition (DIfE), D-14558 Nuthetal, Germany; 3 German Center for Diabetes Research (DZD), D-85764 Munich-Neuherberg, Germany; wenke.jonas@dife.de (W.J.); heikevogel@dife.de (H.V.); tim.schulz@dife.de (T.J.S.); 4 Department of Experimental Diabetology, German Institute of Human Nutrition (DIfE), D-14558 Nuthetal, Germany; 5 Research Group Genetics of Obesity, German Institute of Human Nutrition (DIfE), D-14558 Nuthetal, Germany; 6 Research Group Molecular and Clinical Life Science of Metabolic Diseases, Faculty of Health Sciences, University of Potsdam, D-14369 Potsdam, Germany; 7 Institute of Nutritional Science, University of Potsdam, D-14558 Nuthetal, Germany; 8 Department of Adipocyte Development and Nutrition, German Institute of Human Nutrition (DIfE), D-14558 Nuthetal, Germany; 9 Trace Age-DFG Research Unit on Interactions of Essential Trace Elements in Healthy and Diseased Elderly, Potsdam-Berlin-Jena-Wuppertal, D-14558 Nuthetal, Germany; schwarz.maria@uni-jena.de (M.S.); anna.kipp@uni-jena.de (A.P.K.); 10 Department of Nutritional Physiology, Institute of Nutritional Sciences, Friedrich Schiller University Jena, D-07743 Jena, Germany; 11 Medical Department III-Endocrinology, Nephrology, Rheumatology, University of Leipzig Medical Center, D-04103 Leipzig, Germany; matthias.blueher@medizin.uni-leipzig.de; 12 Helmholtz Institute for Metabolic, Obesity and Vascular Research (HI-MAG) of the Helmholtz Zentrum München at the University of Leipzig, D-04103 Leipzig, Germany

**Keywords:** selenite, insulin, adipose tissue, obesity, insulin resistance

## Abstract

The intake of high-fat diets (HFDs) containing large amounts of saturated long-chain fatty acids leads to obesity, oxidative stress, inflammation, and insulin resistance. The trace element selenium, as a crucial part of antioxidative selenoproteins, can protect against the development of diet-induced insulin resistance in white adipose tissue (WAT) by increasing glutathione peroxidase 3 (GPx3) and insulin receptor (IR) expression. Whether selenite (Se) can attenuate insulin resistance in established lipotoxic and obese conditions is unclear. We confirm that *GPX3* mRNA expression in adipose tissue correlates with BMI in humans. Cultivating 3T3-L1 pre-adipocytes in palmitate-containing medium followed by Se treatment attenuates insulin resistance with enhanced GPx3 and IR expression and adipocyte differentiation. However, feeding obese mice a selenium-enriched high-fat diet (SRHFD) only resulted in a modest increase in overall selenoprotein gene expression in WAT in mice with unaltered body weight development, glucose tolerance, and insulin resistance. While Se supplementation improved adipocyte morphology, it did not alter WAT insulin sensitivity. However, mice fed a SRHFD exhibited increased insulin content in the pancreas. Overall, while selenite protects against palmitate-induced insulin resistance in vitro, obesity impedes the effect of selenite on insulin action and adipose tissue metabolism in vivo.

## 1. Introduction

The obesity pandemic is constantly growing and has become a global health challenge. Although obesity does not always lead to prompt metabolic deteriorations [1], the majority of obese patients suffer from co-morbidities such as hyperglycemia and insulin resistance, which can ultimately lead to type 2 diabetes mellitus. Of particular importance in obese conditions is insulin resistance, the inability of the body to respond adequately to insulin, thereby disallowing normal nutrient flux and storage, regulated glucose uptake in skeletal muscle and lipogenesis in white adipose tissue (WAT), as well as suppression of hepatic glucose production. The intake of high caloric diets with elevated levels of saturated long-chain fatty acids causes multi-tissue insulin resistance through the induction of oxidative stress and activation of several stress responses, leading to activation of serine/threonine kinases, which attenuates the insulin signaling cascade [2]. Moreover, type 2 diabetic patients exhibit reduced insulin receptor (IR) expression in adipose tissue, resulting in decreased signaling due to weakened receptor expression levels [3,4].

Since oxidative stress is a common feature of insulin resistance, research has focused on investigating the potential protective effect of antioxidants against the development of metabolic disorders [5]. However, a fine balance between pro- and antioxidants is crucial to allow proper signaling within a cell. In this regard, it has been shown that (A) H_2_O_2_ is important for insulin signaling [6], (B) excessive use of antioxidants reduces insulin signaling [7], and (C) the production of mild oxidative stress after exercise is important for continued insulin sensitivity in humans [8]. Moreover, short term accumulation of H_2_O_2_ or mitochondrial-derived reactive oxygen species (ROS) induces insulin secretion of pancreatic beta cells [9,10]. However, excessive accumulation of ROS leads to insulin resistance, decreased insulin secretion, and ultimately cell death [11]. Thus, the fine balance of pro- and antioxidants is a key factor in the regulation of insulin sensitivity. In obese conditions, this fine balance is shifted to the side of elevated oxidative stress, which deteriorates cell metabolism and induces low-grade inflammation and insulin resistance. So far, the use of antioxidants has revealed only limited success for the treatment of metabolic disorders, which might be due to the inability to region-specifically counteract excessive oxidative stress [12]. Major antioxidative enzymes are selenoproteins of the glutathione peroxidase (GPx) family. GPx1 is a major H_2_O_2_-detoxifying enzyme in the cytoplasm, while GPx4 is the predominant form found in mitochondria, and GPx3 is secreted and present in the circulation to detoxify excessive H_2_O_2_ levels. Moreover, the trace element selenium is not only a crucial constituent of these enzymes but also exerts, in the form of selenate, insulin-like effects [13]. Thus, the use of dietary selenium species might increase the antioxidative capacity simultaneously at different cellular localizations. However, both too much and too little selenium intake is linked to metabolic alterations [14], underlining the complexity and difficulty of modulating the redox system to improve insulin sensitivity.

We have previously shown that feeding C57BL/6N mice a selenite-enriched high-fat diet (SRHFD) prevented the diet-induced development of insulin resistance and loss of IR expression in adipose tissue, which was linked to elevated local GPx3 levels in WAT [15]. With more than 650 million people suffering from obesity, the question emerges whether selenite supplementation is also able to improve metabolism after established obesity.

In this study, we show that selenite protects against palmitate-induced insulin resistance in 3T3-L1 preadipocytes and enhances adipocyte differentiation in the presence of a surplus of energy. To investigate the protective effect of selenite supplementation in already obese mice, diet-induced obese (DIO) mice received a SRHFD for 12 weeks. Unexpectedly, DIO mice fed a SRHFD exhibit unaltered body weight, glucose tolerance, and insulin sensitivity compared to HFD-fed control mice. SRHFD-fed obese mice showed only a minor increase in mRNA transcripts of selenoproteins in gWAT, with altered adipocyte morphology and smaller adipocytes. However, feeding a SRHFD led to elevated pancreatic insulin levels in obese mice. Overall, while increasing the selenium supply of pre-adipocytes from a deficient to an adequate concentration protects against palmitate-induced insulin resistance in vitro, selenite supplementation of mice with established obesity has no effect on selenoproteins, such as GPx3, or on insulin action and adipose tissue metabolism in vivo.

## 2. Materials and Methods

### 2.1. Animal Studies

Three-week-old male C57BL/6N mice (Charles River Laboratories, Wilmington, MA, USA) were all continuously kept in a 12 h light/dark cycle at a constant temperature of 22 ± 1 °C, had free access to water, and were fed ad libitum. The high-fat diets (HFD and selenium-rich HFD (SRHFD)) used in this study contained 60% of calories from fat and were manufactured by ssniff Spezialdiäten GmbH, Soest, Germany). The selenium contents were determined to be ~230 ng Se/g diet (HFD) and ~670 ng Se/g diet (SRHFD) (see [15] for detailed analysis). We designed a study to tackle the question, whether selenite supplementation after already pre-existing obesity could recapitulate our previous findings, where mice were fed the HFD or SRHFD from the beginning of the study [15]. Thus, from 5 weeks of age, mice were fed the regular HFD for 8 weeks to induce obesity. During week 12 of age, animals were randomly sorted to receive either the same HFD or the SRHFD for further 10 weeks. The insulin tolerance test was performed after 6 weeks of HFD or SRHFD after the diet switch by intraperitoneal injection of insulin (0.75 U/kg bodyweight). For the oral glucose tolerance test after 8 weeks of HFD or SRHFD after the diet switch, animals were starved for 16 h overnight and 2 g glucose/kg bodyweight was administered. Indirect calorimetry to determine energy expenditure and respiratory quotient was performed after 9 weeks of HFD or SRHFD using the PhenoMasterTM (TSE Systems GmbH, Bad Homburg, Germany). Immediately before the calorimetry, nuclear magnetic resonance spectroscopy (EchoMRI^TM^, EchoMRI LLC, Houston, TX, USA) was used to determine body composition.

### 2.2. Cell Culture

White 3T3-L1 preadipocyte cells were kindly given to us by Prof. Dr. Tilman Grune (German Institute of Human Nutrition, Nuthetal, Germany). Published studies in the literature demonstrate that 3T3-L1 cells have a male genotype [16], yet we were unable to determine specific markers of the Y chromosome. This chromosome imbalance has already been described [16].

Cells were cultured in Gibco DMEM GlutaMAX™ (Thermo Fisher Scientific Inc., Waltham, MA, USA) containing 450 mg/dL glucose, 10% low-selenium fetal bovine serum (final concentration of 9.7 nM selenium, labelled condition “C”), 1% penicillin/streptomycin (Thermo Fisher Scientific Inc., Waltham, MA, USA), and 1% pyruvate (Thermo Fisher Scientific Inc., Waltham, MA, USA) at 37 °C and 5% CO2. For selenium treatment, the same medium was supplemented with 200 nM sodium selenite (Na_2_SeO_3_) and labelled condition “Se”. The 3T3-L1 differentiation and Oil-Red-O staining has been described previously [15]. Acute insulin stimulation was performed by adding 100 nM insulin for 5 min after 3 h serum starvation. For lipotoxic assay conditions, palmitic acid (PA) was coupled to bovine serum albumin (BSA) in a saponification reaction in alkaline conditions at a ratio of 2:1 PA:BSA. PA treatment was performed by adding PA:BSA at a final concentration of 250 µM for acute lipotoxic stress conditions or 125 µM for differentiation experiments, respectively, into the media (conditions CPA or SePA). The according BSA concentration was used as an untreated control condition.

### 2.3. Analytical Procedures

Blood glucose was analyzed with a glucometer (Contour XT, Bayer AG, Leverkusen, Germany). ELISA kits from the following manufactures were used: Plasma and pancreatic Insulin Alpco (Alpco, Salem, MA, USA); Leptin, R&D Systems (R&D Systems/Bio-Techne GmbH, Wiesbaden, Germany). The manufacturer’s recommendations were followed. Triacylglycerols (TAGs) were measured with Triglyceride Reagent from ABX. Non-esterified fatty acids (NEFA) were measured with NEFA-HR Assay (FUJIFILM Wako Chemicals Europe GmbH, Neuss, Germany).

### 2.4. RNA Isolation

The ReliaPrepTM RNA Tissue Miniprep System (Promega Corporation, Madison, WI, USA) was used to extract total RNA from murine tissue. For in vitro experiments, we used a phenol–chloroform extraction to isolate total RNA from cultured cells.

### 2.5. Analysis of Gene Expression by Quantitative Real-Time PCR

RNA from tissue or cells was reverse transcribed using oligo(dT)15 primers, random primers, dNTP-Set, and M-MLV reverse transcriptase (Promega Corporation, Madison, WI, USA). Real-time quantitative PCR was performed using GoTaq qPCR master mix (Promega Corporation, Madison, WI, USA) in the CFX384 Touch Real-Time PCR Detection System (Bio-Rad Laboratories Inc., Hercules, CA, USA) and gene-specific primers (Table S1). The expression of genes of interest was calculated by the ΔΔCT method and with *Tbp* (TATA binding protein) as the reference gene. A melting curve analysis confirmed the specificity of the primer pairs.

### 2.6. Western Blot Analysis

Proteins separated by SDS-PAGE were transferred onto PVDF membranes (AmershamTM Hybond^®^, VWR International, LLC, Radnor, PA, USA). Membranes were blocked using StartingBlockTM T20 (TBS) Blocking Buffer (Thermo Fisher Scientific Inc., Waltham, MA, USA). The primary antibodies used to detect proteins of interest are listed in Table S2. Peroxidase-conjugated secondary antibodies are listed in Table S3. Bands were visualized via chemiluminescence using the ChemiDocTM Touch Imaging System (BioRad Laboratories Inc, Hercules, CA, USA). Ponceau staining or β-Actin expression was used as loading control. Densitometric analysis was performed in ImageJ Fiji.

### 2.7. Protein Carbonylation

Carbonylated proteins were detected by derivatization with 20 mM 2,4-dinitrophenylhydrazine (DNPH) in 2 M HCl and subsequent binding to anti-DNP antibody.

### 2.8. Total Reflection X-ray Fluorescence (TXRF)

Fifty milligrams of tissue samples of mice were lysed in RIPA buffer (50 mM Tris (Applichem, Darmstadt, Germany), 150 mM NaCl (Carl Roth, Karlsruhe, Germany), 2 mM EDTA (Applichem), 0.5% sodium deoxycholate (Merck KGaA, Darmstadt, Germany), 0.1% sodium dodecyl sulfate (Applichem), 1% NP-40 (Merck/Millipore, Burlington, MA, USA) containing 1 mg/mL protease inhibitor (Merck/Millipore), followed by two (liver, WAT) or three (kidney) sonification steps (10 × 80% amplitude, 0.5 cycles; Hielscher Ultrasound technology, Teltow, Germany). Afterwards, samples were incubated for 15 min at 4 °C and 1200 rpm using the ThermoMixer^®^ (Eppendorf AG, Hamburg, Germany) and subsequently centrifuged for 10 min at 4 °C and 15,000× *g*. The obtained supernatant was used for trace element analysis with 1 mg/L yttrium (Merck/Millipore) as internal standard. Plasma samples were measured using 1 mg/L gallium (Merck/Millipore) as internal standard. Then, 10 µL of the samples were placed on either siliconized (silicone solution, Serva, Heidelberg, Gemany) (tissue samples) or non-siliconized (plasma) sample carriers and dried at 40 °C. All samples were measured in duplicate for either 1000 s (liver, kidney, plasma) or 1500 s (WAT) using a bench-top total reflection X-ray fluorescence spectrometer (S4 T-STAR, Bruker Nano GmbH, Berlin, Germany). Trace element concentrations of the tissue samples were normalized to protein contents.

### 2.9. Human Studies, Study Participants

The study included 302 individuals (205 women, 97 men; BMI range: 16.9–85.5 kg/m^2^, age range: 16–90 years; for clinical parameters see [15]). Subcutaneous (sc) WAT samples were collected during elective laparoscopic abdominal surgery as described previously and immediately frozen in liquid nitrogen and stored at −80 °C [17,18].

### 2.10. Human Studies, Analysis of GPX3 mRNA Expression in Human WAT

RNA from adipose tissue was extracted using an RNeasy Lipid tissue Mini Kit (Qiagen, Hilden, Germany) and reverse transcribed with standard reagents (Life technologies, Darmstadt, Germany). mRNA expression was performed using TaqMan probes *GPX3:* Hs01078668_m1, *INSR:* Hs00961557_m1) using the QuantStudio 6 Flex Real-Time PCR System (Life technologies, Darmstadt, Germany). Gene expression was calculated using *HPRT1* (hypoxanthine guanine phosphoribosyl transferase 1) as the reference gene.

### 2.11. Statistical Analysis

All data are plotted as means and standard errors of the mean (SEM). We used a two-tailed Student’s *t*-test or two-way ANOVA, respectively, for comparisons between groups. Sidak’s post hoc analysis was included when necessary. A *p*-value cut off to determine significance was set at 0.05. Regression analysis for human data was performed using a linear regression model. GraphPad Prism 7 Software (GraphPad Software Inc., San Diego, CA, USA) was used for statistical analysis and to create graphs.

### 2.12. Data and Resource Availability

All data generated or analyzed during this study are included in the published article (and its online Supplementary Files). No applicable resources were generated during the current study.

## 3. Results

**Figure 1:**
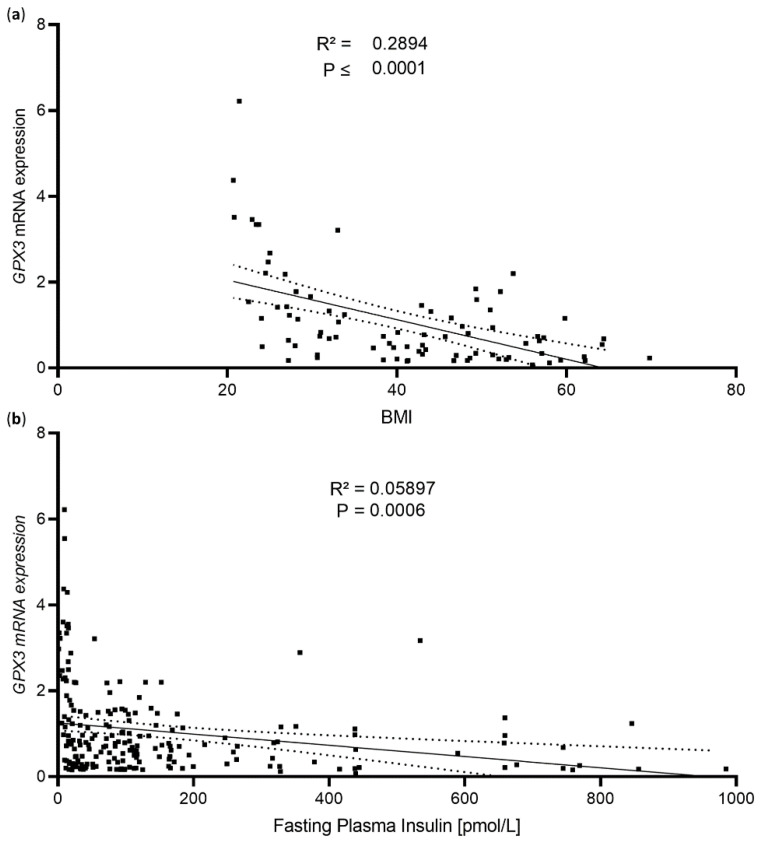
WAT GPX3 expression associates with obesity in humans. (**a**) Correlation of scWAT *GPX3* mRNA expression and patients’ BMIs. (**b**) Correlation of scWAT GPX3 mRNA expression and patients’ fasting plasma insulin levels.

Previously we have shown that (A) the selenoprotein GPx3 regulated IR expression in vitro, (B) *GPX3* expression was lower in overweight/obese patients, and (C) selenite supplementation improved insulin action in WAT [15]. To confirm the link between the selenoprotein GPX3 and obesity and insulin resistance, we analyzed this cohort of obese patients in more detail. Here, *GPX3* mRNA expression in scWAT showed a stronger correlation with BMI than with fasting plasma insulin levels (Figure 1a,b) (for study cohort characteristics see [15]). Interestingly, stratifying the data by sex still showed a significant correlation between BMI and *GPX3* expression in both female (R^2^ = 0.076, *p* ≤ 0.0001) and male (R^2^ = 0.289, *p* ≤ 0.0001) subjects, suggesting a sex-independent effect.

Based on our already published [15] and current data (Figure 1), we raised the question, whether selenite was able to improve insulin signaling and adipose tissue function in already obese conditions. Selenite was specifically chosen as a supplement for the mice, due to its over-the counter availability in terms of human supplements as well as to correspond to our previous study design [15]. In detail, we investigated whether selenite supplementation was able to counteract lipotoxicity-induced insulin resistance in vitro and obesity in vivo.

**Figure 2:**
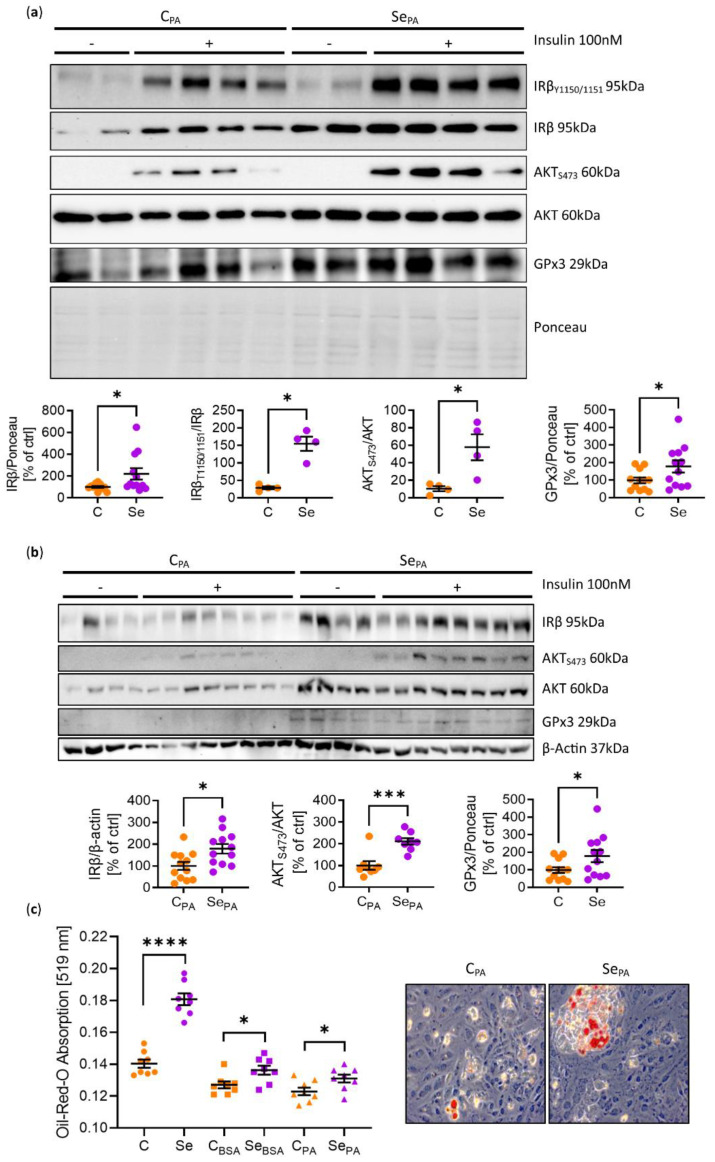
Selenite treatment improves insulin sensitivity under lipotoxic conditions. (**a**) Representative protein phosphorylation, expression, and densitometric analysis of members of the insulin signaling cascade in mature 3T3-L1 adipocytes treated with or without selenite during differentiation and after 100 nM insulin stimulation. (**b**) Representative protein phosphorylation, expression, and densitometric analysis of members of the insulin signaling cascade in 3T3-L1 preadipocytes pre-treated with or without palmitate for three days, with or without selenite for further three days, and after 100 nM insulin stimulation. (**c**) Oil-Red-O absorption and representative staining of 3T3-L1 adipocytes differentiated in the absence or presence of selenite and palmitate for 8 days. *: *p* < 0.05, ***: *p* < 0.001, ****: *p* < 0.0001 after two-tailed Student’s *t*-test. All data are presented as mean ± SEM.

To show that selenite treatment enhanced insulin action and GPx3 expression not only in undifferentiated 3T3-L1 cells [15] but also in mature adipocytes, 3T3-L1 preadipocytes were differentiated in the presence of 200 nM Se for 8 days and acutely treated with 100 nM of insulin for 5 min. Insulin stimulation yielded a five-fold increase in phosphorylation of IR and AKT in 200 nM Se-treated adipocytes compared to low Se-treated (~10 nM) control cells. This was accompanied by an almost three-fold increase in IR protein expression and a 1.7-fold increase in GPx3 expression, confirming the beneficial effect of Se on adipocyte function (Figure 2a). To further assess whether selenite (Se) treatment was able to counteract lipotoxicity-induced insulin resistance—a feature present in metabolically unhealthy obese conditions—3T3-L1 preadipocytes were cultivated for three days in 250 µM palmitate in low-selenium-containing medium. This surplus of energy should mimic lipotoxic conditions and was shown to induce insulin resistance [19]. Subsequently, these cells were co-cultivated in 200 nM containing Se medium for a further three days and insulin-induced phosphorylation of AKT was investigated. Se treatment caused a 450% increase in Ser473 phosphorylation of AKT compared to low-Se-containing control cells, indicating that Se protected against palmitate-induced insulin resistance. Moreover, Se treatment enhanced IR and GPx3 expression, showing that the insulin-sensitizing effect of selenite was also present in lipotoxic conditions (Figure 2b). Next, we assessed the effect of Se treatment in influencing adipocyte differentiation in lipotoxic conditions, as Se treatment enhanced 3T3-L1 adipocyte differentiation in our previous study [15]. Here, differentiation was enhanced by 24% in the presence of elevated palmitate concentrations (Figure 2c).

**Figure 3:**
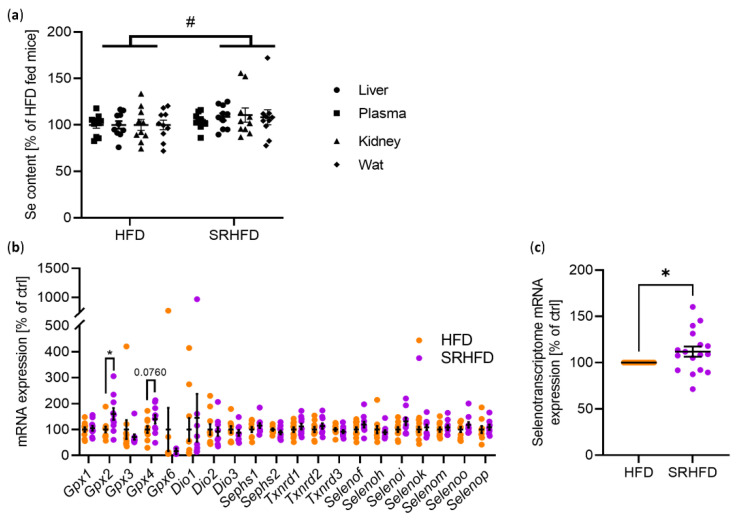
Selenite shows only a mild effect on selenium or selenoprotein contents. (**a**) Total Se content in plasma, liver, kidneys, and WAT, expressed as percent increase relative to the HFD-fed group. (**b**) mRNA expression of selenoproteins in gWAT of male C57BL/6N mice fed either HFD or SRHFD for 10 weeks after established obesity. (**c**) Aggregated selenoprotein mRNA expression from (**b**). *: *p* < 0.05 after two-tailed Student’s *t*-test. #: *p* < 0.05 after two-way ANOVA. All data are presented as mean ± SEM.

To test whether Se treatment also counteracted lipotoxic effects in WAT and improved metabolism after established obesity, we designed the study to first induce obesity in mice by feeding male C57BL/6N mice a HFD (containing 60% of kcal of fat, ~230 ng selenite (Se) per g diet) for 8 weeks. Only males were investigated, as our previous mouse Se-intervention study did not show sex-specific differences [15]. These 12-week-old, diet-induced obese mice with similar elevated blood glucose and plasma insulin levels were evenly distributed into two groups (Figure S1). Half of the mice received the same HFD or were fed a SRHFD containing ~670 ng Se per g diet for an additional 10 weeks. First, we assessed selenium concentrations in the plasma, liver, kidneys, and WAT of HFD- and SRHFD-fed mice. Unexpectedly, we did not find significant increases in plasma or individual tissue selenium content. However, there was an overall significant diet effect observed when calculating selenium contents as percentage increases between the groups (Figure 3a). To test whether this subtle increase in selenium levels was nevertheless able to alter mRNA levels of selenoprotein genes, we determined gene expression of 18 different selenoproteins and *Sephs1* and *Gpx6* in WAT. While only *Gpx2* was significantly increased by 60% in the WAT of mice fed a SRHFD, analysis of all selenoprotein mRNA transcripts revealed a consistent, minor, yet significant 11% increase in total transcript expression (Figure 3b,c), indicating a modest effect of SRHFD feeding of DIO mice.

Mice fed a SRHFD for 10 weeks exhibited unaltered body weight, food intake, and energy expenditure compared to the HFD control (Figure S2a–d). Further, adjusting energy expenditure to either body weight or lean mass using ANCOVA yielded no significant differences between groups. In addition, no difference in blood glucose levels, glucose tolerance, or insulin sensitivity was observed between both groups (Figure S2e–g).

**Figure 4:**
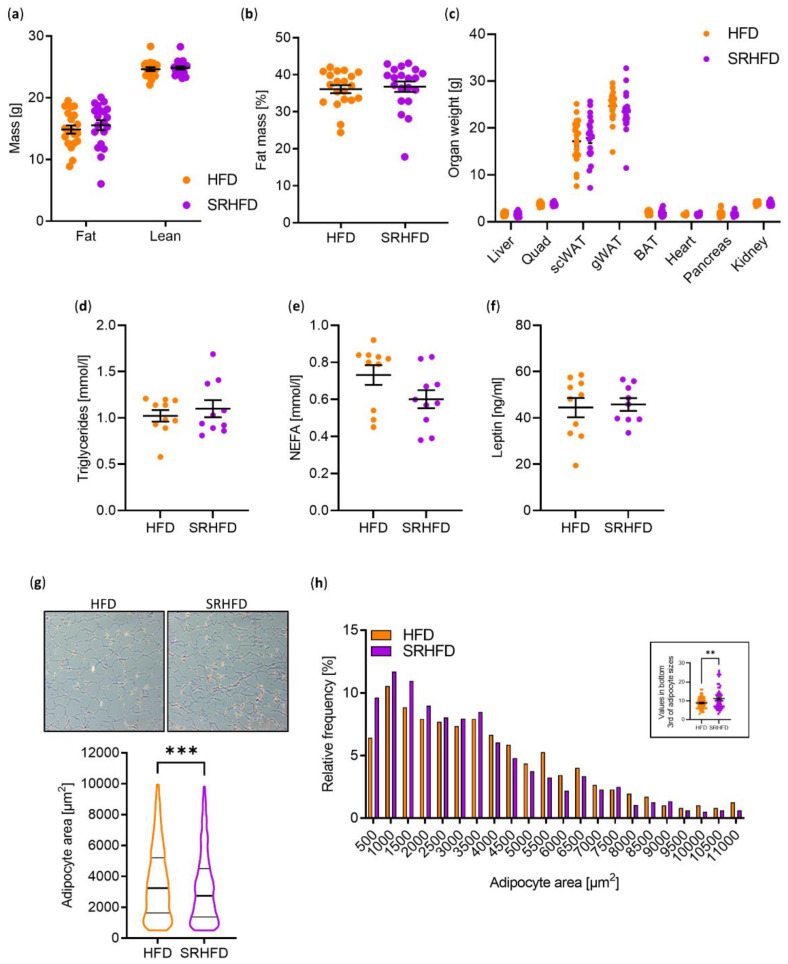
Selenite treatment in established obesity improves adipocyte morphology. (**a**) Body composition measured via NMR of male C57BL/6N mice fed either HFD or SRHFD for 9 weeks after established obesity. (**b**) Body fat percentage calculated from (**a**). (**c**) Final tissue weights of male C57BL/6N mice fed either HFD or SRHFD for 10 weeks after established obesity. (**d**–**f**) Final triglyceride (**d**), non-esterified fatty acids (NEFA, **e**), and leptin (**f**) levels in plasma of male C57BL/6N mice fed either HFD or SRHFD for 10 weeks after established obesity. (**g**,**h**) Morphology, area (**g**), and size distribution (**h**) of gonadal white adipocytes of C57BL/6N mice fed either HFD or SRHFD for 10 weeks after established obesity. **: *p* < 0.01, ***: *p* < 0.001 after two-tailed Student’s *t*-test. All data are presented as mean ± SEM.

Body composition and organ weights of the liver, quadriceps, subcutaneous (sc) WAT, gonadal (g) WAT, brown adipose tissue (BAT), heart, pancreas, and kidneys were indistinguishable between HFD- and SRHFD-fed mice (Figure 4a–c). In line with unaltered WAT weight, plasma triglycerides, non-esterified fatty acids (NEFA), and leptin levels were not changed between groups (Figure 4d–f). However, when analyzing morphological changes in adipocytes, the gWAT of SRHFD fed mice exhibited a smaller adipocyte area, with elevated numbers of small adipocytes in the bottom third of adipocyte sizes (Figure 4g,h). It has been proposed that smaller adipocytes, as observed in our SRHFD-fed group, are metabolically more healthy than larger adipocytes [20].

**Figure 5:**
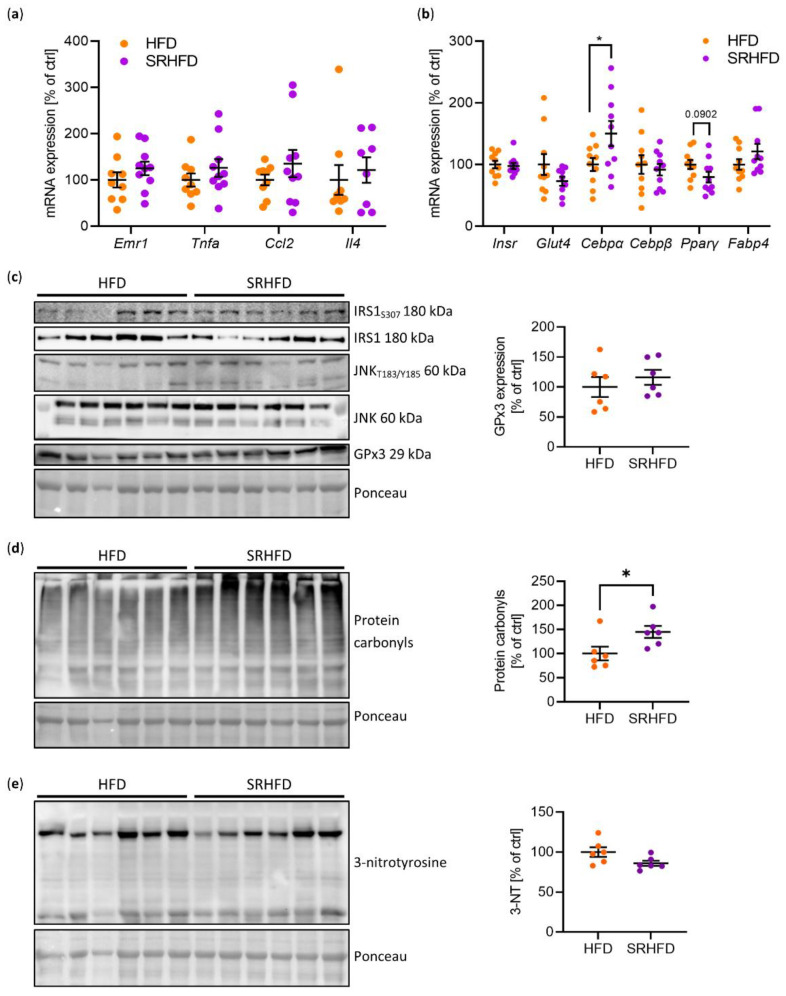
WAT function after selenite treatment in established obesity. (**a**) mRNA expression of inflammatory markers in gWAT of male C57BL/6N mice fed either HFD or SRHFD for 10 weeks after established obesity. (**b**) mRNA expression of adipocyte markers in gWAT of male C57BL/6N mice fed either HFD or SRHFD for 10 weeks after established obesity. (**c**) Protein phosphorylation, expression, and densitometric analysis of insulin resistance markers in gWAT of male C57BL/6N mice fed either HFD or SRHFD for 10 weeks after established obesity. (**d**) Protein carbonylation and densitometric analysis in gWAT of male C57BL/6N mice fed either HFD or SRHFD for 10 weeks after established obesity. (**e**) Protein 3-nitrotyrosine modification and densitometric analysis in gWAT of male C57BL/6N mice fed either HFD or SRHFD for 10 weeks after established obesity. *: *p* < 0.05 after two-tailed Student’s *t*-test. All data are presented as mean ± SEM.

Neither inflammatory markers, such as *Emfr1* (coding for F4/80), *Tnfα*, *Ccl2*, or *Il4*, were changed on the transcript level (Figure 5a), which has been shown to improve insulin sensitivity [21]. Unexpectedly, expression levels of *Gpx3* and *Insr* were unaltered in WAT samples of SRHFD-fed mice (Figure 3b and Figure 5b), suggesting that SRHFD was not sufficient to increase GPx3 expression, which is necessary to enhance IR expression. Only *Cebpα* gene expression was significantly increased in WAT samples of SRHFD-fed mice, while terminal differentiation markers such as *Fabp4* remained unchanged (Figure 5b).

Next, we investigated whether the WAT of SRHFD-fed mice revealed a molecular signature of improved insulin sensitivity by assessing negative markers of insulin signaling: IRS1Ser307 phosphorylation and activation (Thr183/Tyr185 phosphorylation) of the ser/thr kinase c-Jun kinase (JNK). No difference in the phosphorylation status of IRS1 or JNK could be observed between tested groups, indicating unaltered insulin sensitivity and inflammation in WAT in SRHFD animals (Figure 5c). Interestingly, protein carbonylation was increased by 45% in WAT samples of SRHFD-fed mice (Figure 5d). As protein carbonylation is a sign of oxidative stress, we further determined a marker of nitrosative stress, 3-nitrotyrosine (3-NT), and found that 3-NT levels remained unchanged between both groups (Figure 5e), indicating only the presence of mild oxidative stress in WAT of SRHFD, which does not lead to enhanced inflammation but is rather linked to an improved WAT morphology (Figure 4g,h and Figure 5a,b).

Thr183/Tyr185 phosphorylation of JNK, as well as GPx1 and IR expression, were also unchanged in the scWAT, liver, and skeletal muscle of SRHFD-fed mice compared to HFD control (Figure S3), revealing an exclusive, modest Se-induced effect on gWAT physiology even though gWAT Se-sensitive selenoprotein expression was unaffected by the Se supply.

**Figure 6:**
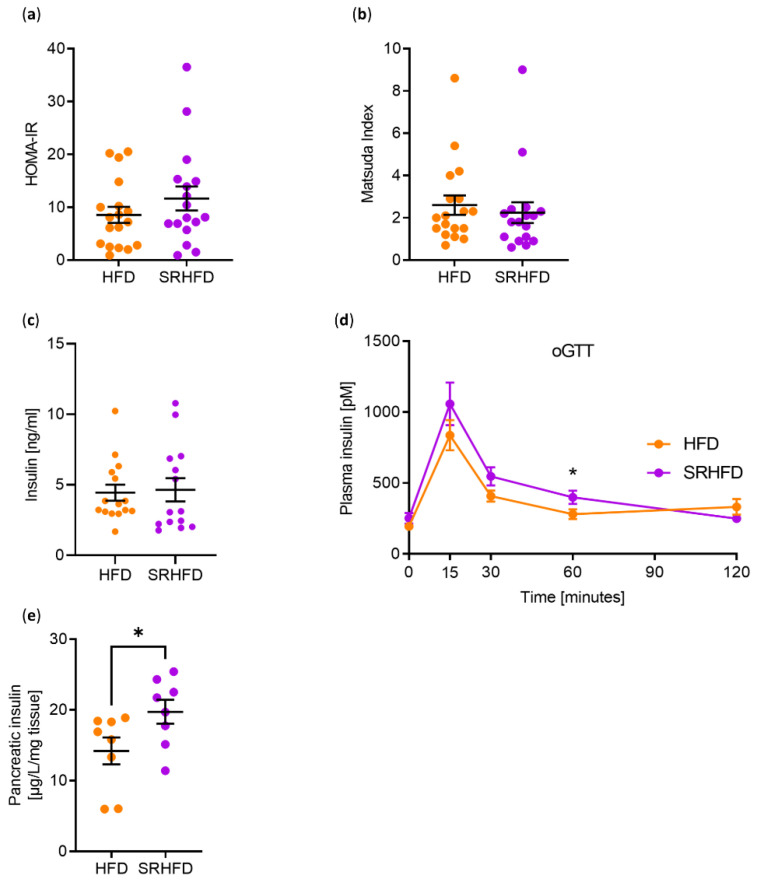
Selenite treatment in established obesity increases pancreatic insulin content. (**a**) Calculated values for the homeostatic model assessment of insulin resistance HOMA-IR using data from the oGTT of male C57BL/6N mice fed either HFD or SRHFD for 8 weeks after established obesity. (**b**) Calculated values for the Matsuda insulin sensitivity index using data from the oGTT of male C57BL/6N mice fed either HFD or SRHFD for 8 weeks after established obesity. (**c**) Plasma insulin levels of male C57BL/6N mice fed either HFD or SRHFD for 10 weeks after established obesity. (**d**) Plasma insulin levels during the oGTT of male C57BL/6N mice fed either HFD or SRHFD for 8 weeks after established obesity. (**e**) Pancreatic insulin levels of male C57BL/6N mice fed either HFD or SRHFD for 10 weeks after established obesity. *: *p* < 0.05 after two-tailed Student’s *t*-test. All data are presented as mean ± SEM.

To gain more insights into potential alterations in insulin sensitivity, we calculated the HOMA-IR (Homeostatic Model of Insulin Resistance) and the Matsuda insulin sensitivity index. Here, HFD- and SRHFD-fed mice revealed similar insulin resistance (HOMA-IR, HFD = 8.6 vs. SRHFD = 11.7, *p* = 0.258) and insulin sensitivity scores (Matsuda Index, HFD = 2.6 vs. SRHFD = 2.3, *p* = 0.5972) (Figure 6a,b).

We further investigated whether Se supplementation was able to alter insulin release from the pancreas, since it has been shown that selenite induced beta-cell gene expression and enhanced islet function in vitro [22]. Insulin levels between mice fed ad libitum with either HFD or SRHFD, respectively, were indistinguishable (Figure 6c). Next, we investigated insulin release during the performed oGTT (Figure S2f). There was no overt difference in insulin release during the oGTT, but a significant increase in plasma insulin levels after 60 min during the glucose tolerance test (Figure 6d). This does not seem to have been due to altered insulin stability, as mRNA expression of insulin-degrading enzyme (IDE) was indistinguishable between both groups in all tested organs (Figure S4). Interestingly, SRHFD-fed mice exhibited 38% increased pancreatic insulin levels, suggesting that selenite enhances insulin production and tends to enhance insulin release (Figure 6e).

Overall, we have shown that selenite protects against lipotoxicity-induced insulin resistance and adipocyte dysfunction in vitro, while selenite supplementation after established obesity exerts only a modest effect on adipocyte morphology and enhances insulin production in the pancreas.

## 4. Discussion

Obesity is a risk factor for the development of cardiovascular diseases, certain cancers, and metabolic disorders such as type 2 diabetes. A surplus of energy in the form of free fatty acids and lipids is stored in adipose tissue but can also lead to insulin resistance [19]. Insulin resistance is linked to oxidative stress and adipocyte dysfunction and can worsen overall metabolism. Moreover, type 2 diabetes is characterized by decreased IR expression in WAT [4], demonstrating that reduced insulin sensitivity with decreased IR expression is tightly linked to adipocyte dysfunction. We have previously shown that the selenoprotein GPx3 was not only decreased in long-term insulin-resistant conditions but also linked to IR expression in adipocytes [15]. Thus, decreasing GPx3 expression caused insulin resistance in 3T3-L1 preadipocytes with decreased IR expression. Conversely, feeding lean mice a SRHFD to enhance selenoprotein synthesis protected against the development of diet-induced insulin resistance and increased GPx3 and IR expression in WAT. While this demonstrates that selenite protects against the development of obesity in mice, it is unclear whether selenite can counteract established insulin resistance in obesity.

In this study, we have shown that selenite improves insulin sensitivity in differentiated 3T3-L1 adipocytes (Figure 2a), a phenotype already observed in undifferentiated cells [15]. Thus, Se treatment improves overall adipocyte differentiation and function, which supports data showing that Se regulates genes involved in lipid metabolism [23]. Our study reveals that Se also increased the differentiation and function of 3T3-L1 adipocytes even in the presence of elevated palmitate levels, which in general induces insulin resistance in 3T3-L1 pre-adipocytes (Figure 2b). These data demonstrate that Se treatment in comparison to Se deficiency counteracts negative effects of lipotoxic metabolites in vitro. However, Se supplementation was unable to recover insulin signaling in the WAT of already obese mice. As Se induced GPx3 and IR in vitro (Figure 2a,b) but not in vivo (Figure 3b and Figure 5b), these data point to a crucial role for GPx3 in improving adipocyte function (Figure 2a–c). Indeed, in our current (Figure 1) and previous study (Hauffe et al. [15]), we have identified a positive correlation between *GPX3* expression in subcutaneous white adipose tissue and markers of insulin sensitivity in humans, supporting the hypothesis that the upregulation of GPX3 exerts beneficial effects in adipose tissue. Interestingly, this effect seems to be sex-independent, as this significant correlation remained even after stratifying the data in both female and male subcohorts. As this naturally decreased the number of participants in the available study population, this clearly needs further validation in a larger cohort of human participants. Moreover, GPX3 belongs to an adipokine cluster related to insulin sensitivity/hyperglycemia and lipid metabolism in humans [24]. Thus, the inability of selenite supplementation to enhance GPx3 in established obesity can explain why obesity prevents a supplemented Se-induced insulin-sensitizing effect in adipose tissue.

While feeding lean mice a SRHFD caused adipocyte hypertrophy and improved insulin sensitivity in WAT [15], feeding DIO mice a SRHFD resulted in smaller adipocyte area, indicative of adipocyte hyperplasia, and unaltered insulin sensitivity. The reasons for this discrepancy remain unknown but might relate to differences in selenium status or selenoprotein gene expression in the tissue. The WAT of DIO mice fed a SRHFD exhibited only an upregulation of *Gpx2* and a minor yet significant upregulation of all analyzed selenoprotein mRNA levels (Figure 3c). In contrast, in our “preventive” selenite supplementation study, mRNA levels of all selenoprotein genes were unchanged between diets (HFD set to 100%, SRHFD 104%, *p* = 0.47 [15]). Thus, an overt increase in gene expression of selenoproteins cannot account for the observed difference between studies. Yet, *Gpx1*, *Gpx3*, *Sephs2*, and *Txnrd3* mRNA levels were significantly increased in the “preventive” selenite supplementation study [15]. As GPx1 KO mice fed an obesogenic diet show decreased adiposity with unaltered insulin sensitivity [25] and GPx1 overexpression causes obesity and insulin resistance [26], GPx1 function in adipose tissue might not explain the differences between studies. Again, *GPX3* seems to be important in this aspect regarding human physiology [24,27], while *SEPHS2* and *TXNRD3* expression are likely of minor importance as their expression is unaltered between lean and obese patients [15].

It needs to be stated that all the mice in our study (HFD- and SRHFD-fed) received supraphysiological selenium concentrations through the diet [28,29]. This concentration was chosen to ensure that the control group did not develop selenium deficiency, which would artificially inflate any results obtained. Simultaneously, we aimed for a supplementation regime that would not induce metabolic deteriorations, as this can occur with selenium oversupply [14]. Thus, only a small increase in selenium concentrations was expected, as was already observed in our previous study [15]. Our data indicate that selenite uptake is aggravated in established obesity compared to lean mice that receive a SRHFD [15]. We were unable to determine single differences in selenium concentrations assessed by Total Reflection X-ray Fluorescence (TXRF) in plasma and tested organs. Based on this, we speculate that intestinal selenite absorption might be reduced in DIO in comparison to lean mice fed the SRHFD. It has been suggested that selenite uptake takes place via simple diffusion across the plasma membrane [30]. As HFD can alter plasma membrane composition [31], this might result in altered Se uptake. Furthermore, palmitate treatment of enterocytes alters cell metabolism, indicating a detrimental effect on intestinal function [32]. In addition, selenium transport from the liver to other organs takes place via SELENOP, which binds to Apoer2 on target cells. This process is potentially modulated by circulating amounts of lipoproteins or triglycerides. Clearly, further research is needed to investigate a potential altered regulation of Se homeostasis in lean and obese conditions.

Why the gWAT of SRHFD-fed mice exhibited increased protein carbonylation, a sign of oxidative stress, but unaltered 3-NT levels is unclear. Of note, such a discrepancy has already been observed in human samples [33]. Adipose tissue exhibits unique carbonylated proteins in obesity and insulin resistance, including GPx1 [34]. Thus, the observed SRHFD-induced effect on protein carbonylation might alter selenoprotein metabolism and insulin signaling in various ways.

Interestingly, SRHFD caused increased insulin levels in the pancreas and tended to enhance insulin release during a glucose tolerance test. This is in line with data showing that selenite induced insulin expression in vitro and release in islets of Langerhans [22]. Mechanistic insights are missing but this might relate to altered SelenoK expression, which regulates insulin secretion in Min6 cells [35]. Alternatively, increased pancreatic but unchanged circulating insulin levels might suggest increased insulin degradation via insulin-degrading enzyme (IDE). However, analyzing mRNA expression of *Ide* in a variety of tissues (gWAT, hypothalamus, liver, quadriceps, and kidney) shows no differences in *Ide* expression, suggesting that IDE-mediated degradation of insulin does not play a major role in our observed phenotype (Figure S4).

In our present study, we only investigated male mice, as there was no sex-specific difference after selenite supplementation in our previous study. Of note, stratifying our human data by sex also revealed significant correlations between *GPX3* expression and BMI in both female and male cohorts individually. However, considering the differences in results between the studies, sex-specificity cannot be ruled out in the current research design. Given the intricate involvement of sex hormones in metabolism, it is difficult to predict what the phenotype of female mice would be in our research design. Clearly, further research is warranted to analyze this aspect.

The correlation between adipose *GPX3* mRNA expression and BMI in humans further highlights that elevated *Gpx3* levels correlated with a lean phenotype in mice. Moreover, the weak yet significant association between *GPX3* and fasting plasma insulin indicates that GPX3 expression is crucial for glucose homeostasis and insulin sensitivity. As DIO mice fed a SRHFD do not exhibit altered GPx3 levels compared to HFD-fed control mice, the absence of overall improved insulin signaling in WAT as well as unaltered global glucose tolerance and insulin sensitivity in the Se-supplemented group is therefore scientifically not unexpected. Additional research needs to focus on understanding how GPx3 expression in adipose tissue can be regulated even in selenium-adequate conditions. Since GPx3 expression in WAT was reduced in db/db compared to db/+ mice that received the same diet [15], other factors can modulate GPx3 expression. Finding these factors might help to counteract diet-induced insulin resistance in such conditions.

## 5. Conclusions

In summary, we have shown that selenite protects against palmitate-induced insulin resistance with increased GPx3 and IR expression in vitro, while the selenium intervention was not successful in modulating plasma selenium status and GPx3 expression under conditions of obesity in vivo. Accordingly, no selenite-induced improvement in insulin sensitivity, with only a modest effect on adipocyte morphology and enhanced insulin production in the pancreas, was observed. Whether these marginal metabolic effects are due to lesser selenite uptake in obese conditions warrants further investigation. Our data might also imply that higher selenite concentrations are needed to profoundly improve metabolism in established obesity.

## Data Availability

All data generated or analyzed during this study are included in the published article (and its online Supplementary Files). No applicable resources were generated during the current study.

## Supplementary Materials

The following supporting information can be downloaded at: https://www.mdpi.com/article/10.3390/antiox11050862/s1, Figure S1: Characteristics of mice before SRHFD intervention, Figure S2: Selenite treatment in established obesity does not improve overall metabolic phenotype, Figure S3: Tissue expression of stress marker and selenoproteins, Figure S4: Tissue expression of insulin-degrading enzyme (*Ide*) mRNA, Table S1: Real-time quantitative PCR primer pairs, Table S2: Primary antibodies for Western blotting, Table S3: Secondary antibodies for Western Blotting.
