# Supplementary material for: Obesity Hinders the Protective Effect of Selenite Supplementation on Insulin Signaling
Source: Antioxidants (Basel). 2022 Apr 28;11(5):862. doi: 10.3390/antiox11050862 (PMC9138114; doi:10.3390/antiox11050862)
Supplement: Supplementary file 1 [file antioxidants-11-00862-s001.zip › antioxidants-1685565-supplementary.pdf]

Obesity impedes the protective effect of selenite supplementation on insulin signaling.

Robert Hauffe, Michaela Rath, Wilson Agyapong, Wenke Jonas, Heike Vogel, Tim J. Schulz, Maria Schwarz, Anna P. Kipp, Matthias Blüher, and André Kleinridders

Supplementary Figures and Material

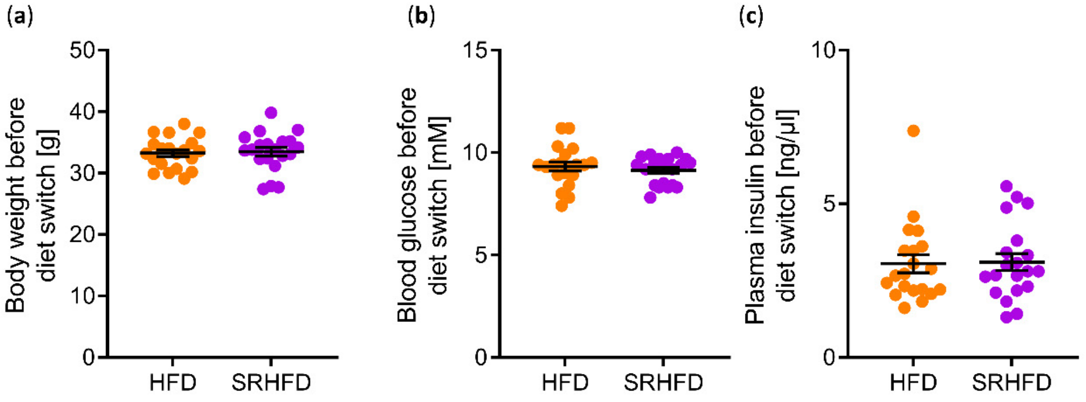

Figure S1. Characteristics of mice before SRHFD intervention. (**a**) Body weight of male C57Bl/6N mice fed HFD for 8 weeks. (**b**) Blood glucose levels of male C57Bl/6N mice fed HFD for 8 weeks. (**c**) Plasma insulin of male C57Bl/6N mice fed HFD for 8 weeks. All data are presented as mean ± SEM.

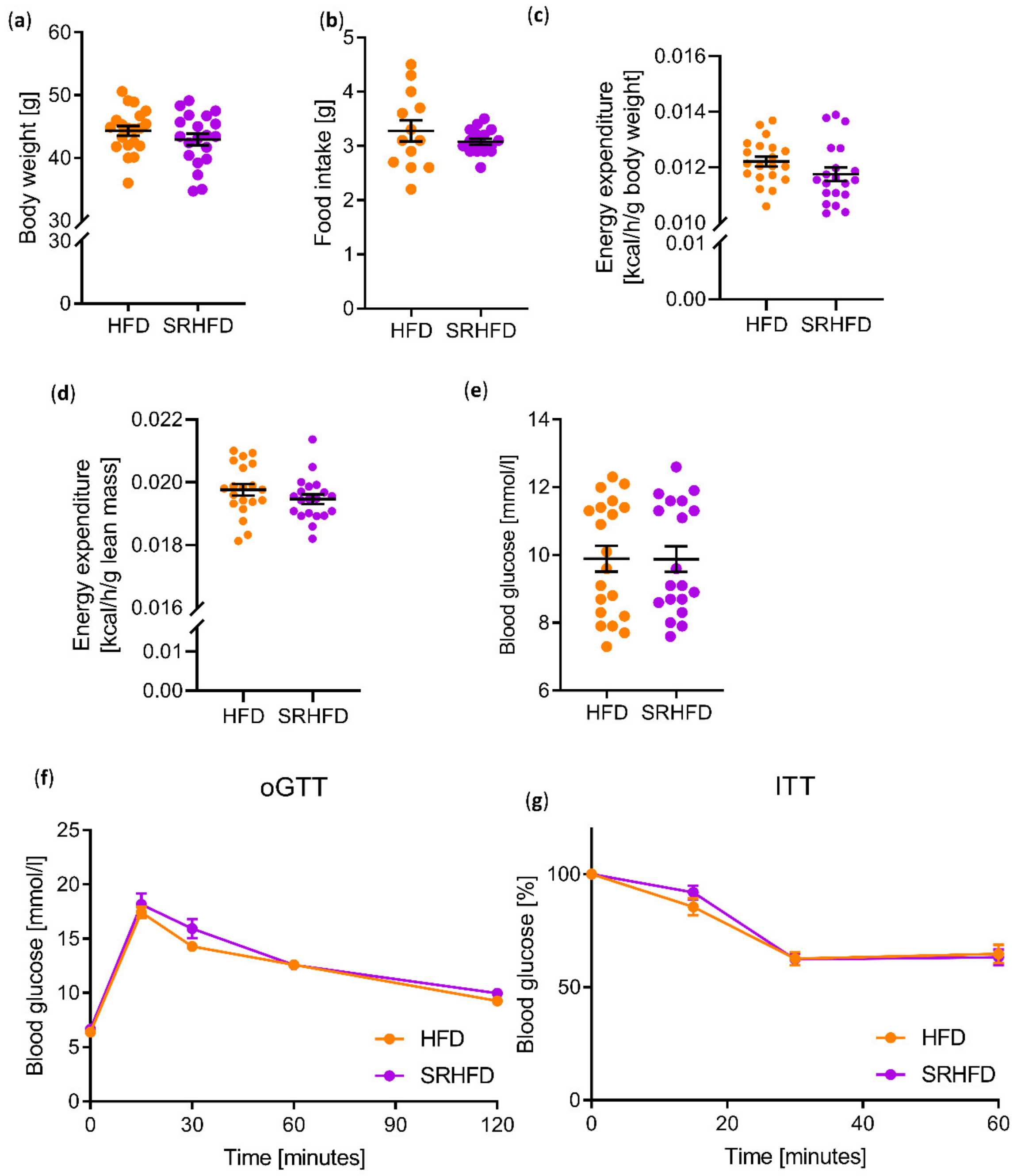

Figure S2. Selenite treatment in established obesity does not improve overall metabolic phenotype. (**a**) Final bodyweight of male C57Bl/6N mice fed either HFD or SRHFD for 10 weeks after established obesity. (**b**) Food intake of male C57Bl/6N mice fed either HFD or SRHFD for 9 weeks after established obesity. (**c, d**) Energy expenditure relative to body weight (c) or lean mass (d), respectively, of male C57Bl/6N mice fed either HFD or SRHFD for 9 weeks after established obesity. (**e**) Random fed blood glucose levels of male C57Bl/6N mice fed either HFD or SRHFD for 10 weeks after established obesity. (**f**) Blood glucose levels during an oGTT of male C57Bl/6N mice fed either HFD or SRHFD for 8 weeks after established obesity. (**g**) Blood glucose levels during an ITT of male C57Bl/6N mice fed either HFD or SRHFD for 6 weeks after established obesity. All data are presented as mean ± SEM.

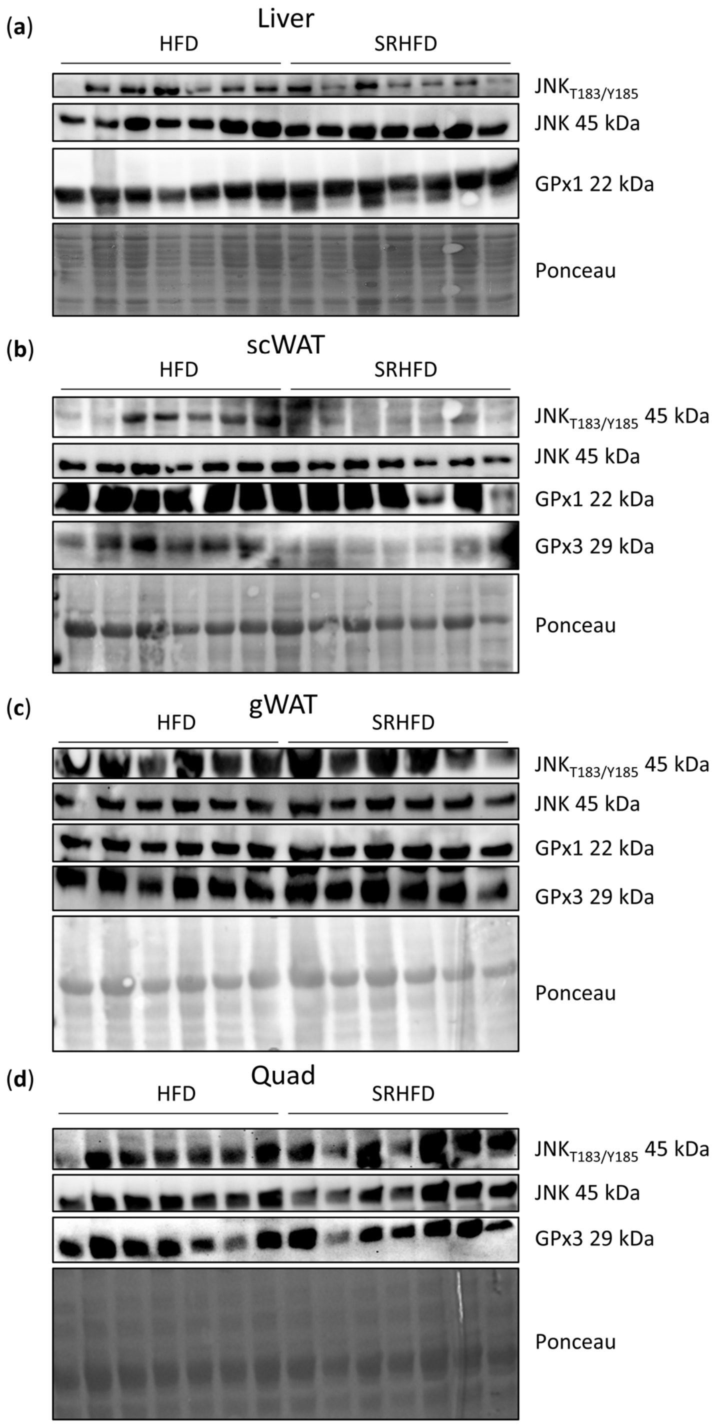

Figure S3. (**a-d**) Protein phosphorylation, expression and densitometric analysis of stress markers and selenoproteins in liver (a), gWAT (b), scWAT (c), and quadriceps (d) of male C57Bl/6N mice fed either HFD or SRHFD for 10 weeks after established obesity.

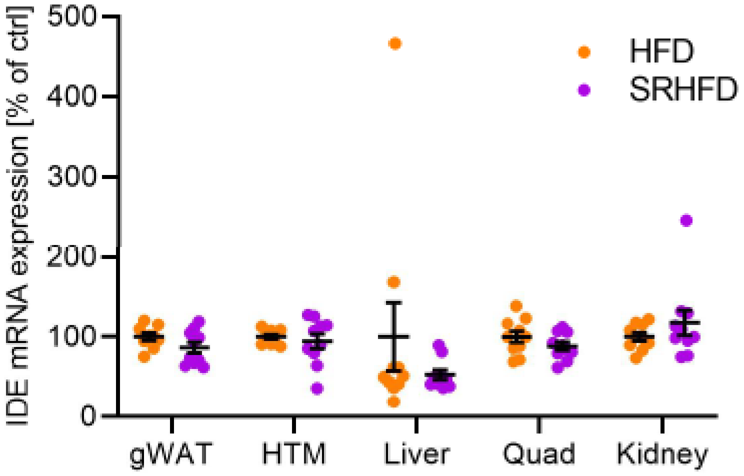

Figure S4. Tissue expression of insulin degrading enzyme (Ide) mRNA. Gene expression analyzed om tissues of male C57Bl/6N mice fed either HFD or SRHFD for 10 weeks after established obesity. All data are presented as mean ± SEM.

Table S1: Real-time quantitative PCR primer pairs

**Target gene**	**Forward sequence**	**Reverse sequence**
Tbp (TATA-binding-protein	CTGGAATTGTACCGCAGCTT	ATGATGACTGCAGCAAATCG
Pppary (Peroxisome proliferator-activated receptor gamma)	CCCAGAGCATGGTGCCTTCGC	AGTTGGTGGGCCAGAATGGCA
Ap2 (Adipocyte protein 2)	AAGGTGAAGAGCATCATAACCCT	TCACGCCTTTCATAACACATTCC
Cebpa (CCAAT/enhancer-binding protein alpha)	CAAGAACAGCAACGAGTACCG	GTCACTGGTCAACTCCAGCAC
Cebpb (CCAAT/enhancer-binding protein beta)	CAAGCTGAGCGACGAGTACA	AGCTGCTCCACCTTCTTCTG
Glut4 (Glucose transporter type 4)	CATTCCCTGGTTCATTGTGG	GAAGACGTAAGGACCCATAGC
Ir (Insulin receptor)	AATGGCAACATCACACACTACC	AATGGCAACATCACACACTACC
Gpx1 (Glutathione peroxidase 1)	ACAGTCCACCGTGTATGCCTTC	CTCTTCATTCTTGCCATTCTCCTG
Gpx2 (Glutathione peroxidase 2)	GTGCTGATTGAGAATGTGGC	AGGATGCTCGTTCTGCCCA
Gpx3 (Glutathione peroxidase 3)	CCATTTGGCTTGGTCATTCTGGG	CACCTGGTCGAACATACTTGAGAC
Gpx4 (Glutathione peroxidase 4)	TCTGTGTAAATGGGGACGATGC	TCTCTATCACCTGGGGCTCCTC
Gpx6 (Glutathione peroxidase 1)	GCACATCCTCTTTGTCAACG	CTTTCCAGGTTCTTGCTTTCC

Txnrd1 (Thioredoxin reductase 1)	ATGAGAATGCTTACGGGAGGT	GGAACCGCTCTGCTGAATAGAT
Txnrd2 (Thioredoxin reductase 2)	GATCCGGTGGCCTAGCTTG	TCGGGGAGAAGGTTCCACAT
Txnrd3 (Thioredoxin reductase 3)	CGACAACGAACGTGTGGTGG	AGTAGCTGCTTCGTGAGCCC
Dio1 (Iodothyronine deiodinase 1)	AGAGACTCGTAGATGACTTTGCC	GCCGGATGTCCACGTTGTT
Dio2 (Iodothyronine deiodinase 2)	TTTGATGTGTCAGGAGTCGGG	CCAACATTCCCTACCCCAAGA
Dio3 (Iodothyronine deiodinase 3)	CACGGCCTTCATGCTCTGG	CGGTTGTCGTCTGATACGCA
Sephs1 (Selenophosphate synthetase 1)	TGAACTGAAAGGCACAGGCTGC	CGCAAGTATCCATCCCAATGC
Sephs2 (Selenophosphate synthetase 2)	ACCGACTTCTTTTACCCCTTGG	TCACCTTCTCTCGTTCCTTTTCAC
Sep15 (15 kDa selenoprotein)	GTTTCAAGCGGCGTCTGCTC	TGCTTCTTCCTGACAGCACCC
SelenoH (Selenoprotein H)	CCTTATTCCACCAACGCGCCA	GCGTCAGCTCGTACAATGCTC
SelenoK (Selenoprotein K)	ATGGAAGAGGGCCACCAGGA	TTACCTTCCTCATCCACCAGCC
SelenoI (Selenoprotein I)	ACTGGTTACTGCTTCCTCTCCTC	CTGCTTCACCACTTGTACGCC
SelenoM (Selenoprotein M)	CGGATTGGAACCGTCTTCGAG	CACCTCCTTTAGGCGATTCAAC
SelenoO (Selenoprotein O)	TGACACTGAGTTCCAAAGGCAC	GTTAGTGAAGTCAGCACCAGTCAG
SelenoP (Selenoprotein P)	CCTTGGTTTGCCTTACTCCTTCC	TTTGTTGTGGTGTTTGTGGTGG
SelenoS (Selenoprotein S)	CAGAAGATTGAAATGTGGGACAGC	CCTTTGGGGATGACAGATGAAGTAG
SelenoT (Selenoprotein T)	CTTTAAATGATGTGCCAGTGTGGT	GGTAGGGCTATGATCGATGATGTG
SelenoV (Selenoprotein V)	CCCAACAGAATCTTGATCCGTG	TTCAAACTCCCCTGTAACCTG
SelenoW (Selenoprotein W)	GCCGTTCGAGTCGTGTATTGT	CACTTCAAAGAACCCGGTGAC
SelenoX (Selenoprotein X)	ACTTCGAGCCAGGTGTCTACG	GGCACTTGGTCACACTGTCTG
F4/80 (EGF-like module-containing mucin-like hormone receptor-like 1)	GAATCTTGGCCAAGAAGAGAC	GAATTCTCCTTGTATATCATCAGC
Tnfα (Tumor necrosis factor alpha)	CTTCTGTCTACTGAACTTCGGG	CAGGCTTGTCACTCGAATTTTG
Ccl2 (chemokine (C-C motif) ligand 2)	GTCCCTGTCATGCTTCTGG	GCTCTCCAGCCTACTCATTG
Il-4 (Interleukin 4)	ACAGGAGAAGGGACGCCAT	GAAGCCCTACAGACGAGCTCA

Table S2: Primary antibodies for Western Blotting

Antigen	Company	Cat.-Nº.
3-Nitrotyrosine (3-NT)	Abcam plc.	ab110282
β-Actin	Santa Cruz	sc-47778
AKT	Cell Signaling Technology, Inc	9272
AKT_{Ser473}	Cell Signaling Technology, Inc	9271
DNP (Carbonylation)	Merck KGaA	D9656
GPx1	Abcam plc.	ab22604
GPx3	Thermo Fisher Scientific, Inc.	PA5-18677
IRβ	Cell Signaling Technology, Inc	3025
$IR\beta_{Y1150/1151}$	Santa Cruz	sc-81500
IRS1	Cell Signaling	#2382
$IRS1_{S307}$	Cell Signaling	#2381
JNK	Cell Signaling	#9252
$JNK_{T183/Y185}$	Cell Signaling	#9251

Table S3: Secondary antibodies for Western Blotting

Species	Company	Cat.-Nº.
Rabbit	Cell Signaling Technology, Inc	7074S
Mouse	Cell Signaling Technology, Inc	7076S
Goat	Jackson Immuno Research, Inc.	305-035-006
